# Fear of COVID-19, traumatic growth, and traumatic stress symptoms: the mediating role of basic psychological needs

**DOI:** 10.3389/fpsyg.2024.1440001

**Published:** 2025-01-23

**Authors:** Rafael Gargurevich, Valeria Campodónico, Lennia Matos

**Affiliations:** Department of Psychology, Pontifical Catholic University of Peru, Lima, Peru

**Keywords:** fear of COVID-19, basic psychological needs, post-traumatic growth, traumatic stress symptoms, Peruvian community sample

## Abstract

Although COVID-19 brought many negative psychological consequences, many people also experienced psychological growth. The present study investigated why this happened and hypothesized that self-determination theory’s (SDT’s) basic psychological needs (BPNs) may play a role in this explanation. Based on SDT, this cross-sectional study hypothesized that both the satisfaction and frustration of BPNs mediated the associations between fear of COVID-19, post-traumatic growth (PTG), and post-traumatic stress (PTS) symptoms. A sample of 391 Peruvian adults (70.6% women; *M_age_* = 35.04, age range between 18 and 84) responded to several valid and reliable questionnaires: Fear of COVID-19 Scale (FCV.19S); Basic Psychological Needs Satisfaction and Frustration Scale (BPNSFS); Impact Event Scale-Revised (IES-R); Post-Traumatic Growth Inventory (PTGI). The results showed that need satisfaction mediated the negative association between fear of COVID-19 and PTG, and need frustration mediated the positive association between fear of COVID-19 and PTG. In addition, a direct effect was found from fear of COVID-19 toward PTS symptoms. The results showed the importance of considering SDT’s BPNs in explaining PTG and PTS after experiencing fear of COVID-19.

## Introduction

In 2019, the outbreak of SARS-CoV-2, also known as COVID-19, spread out around the world causing a global pandemic. In January 2020, the World Health Organization (WHO) declared a global health emergency (Velavan and Meyer, 2020), and in March 2020, the COVID-19 virus arrived in Peru (MINSA, 2020). Given the precarious health system (Gianella et al., 2021) and despite the efforts to prevent contagions (e.g., using masks and care few), Peru was one of the countries with the highest mortality rates because of the COVID-19 with 221,078 deaths (MINSA, 2024; Johns Hopkins University Coronavirus Resource Center, 2023).

The COVID-19 pandemic caused many negative consequences in the emotional, behavioral, cognitive, and physical aspects of individuals (Macaya and Aranda, 2020; MINSA, 2020; Hawes et al., 2022; Taheri et al., 2023). The pandemic generated feelings of anxiety, helplessness, frustration, fear, guilt, irritability, sadness, emotional distancing, avoidance of certain environments situations or people, difficulties in disconnecting from work, and changed the work and family environments also causing many interpersonal problems (Bertollo et al., 2023; Macaya and Aranda, 2020; Maguiña, 2020; Pedrosa et al., 2020; Ritchie et al., 2020).

Given the high mortality rates in the country due to COVID-19 throughout the pandemic in Peru (Karlinsky and Kobak, 2021), fear of dying by the virus was quite prevalent (Sotomayor-Beltran et al., 2021: Ramos et al., 2022). In fact, a large part of the negative impact of COVID-19 on mental health may be due to the fear of dying by the virus, also described as fear of COVID-19 (Fiorillo and Gorwood, 2020).

Fear is an unpleasant emotional state that is generated by the perception of a threatening stimulus (de Hoog et al., 2008). So, situations such as natural disasters, the outbreak of a disease, or epidemics can lead to fear in many people (Pakpour and Griffiths, 2020). As such, fear of COVID-19 has been related to depressive and anxiety symptomatology (Fitzpatrick et al., 2020) and even suicide (Goyal et al., 2020; Mamun and Griffiths, 2020), and it has generated symptoms of post-traumatic stress (PTS; Fiorillo and Gorwood, 2020; Tomaszek and Muchacka-Cymerman, 2020; Chen et al., 2020) but remarkably also signs of post-traumatic growth (PTG; Chen and Tang, 2021; Jian et al., 2023, Bonazza et al., 2022).

Post-traumatic growth can be defined as a positive psychological change experienced because of a struggle with highly challenging and stressful life circumstances (Tedeschi et al., 1998). This concept explains how certain people who, despite having been exposed to a traumatic event, do not necessarily develop negative psychological sequelae (such as maladaptive behaviors or disorders) but manage to develop adaptive reactions (Calhoun and Tedeschi, 2006). The word “growth” within this concept arises because people experiencing this phenomenon have developed new levels of adaptation, psychological functioning, or a new awareness of life in a way that they did not have before (Taku et al., 2008; Tedeschi et al., 2018). So, stressful fearful circumstances could generate individuals to have more meaningful interpersonal relationships, have a greater sense of personal strength, change priorities in life, have a greater appreciation for smaller life events, or have a more existentially or spiritually richer life (Tedeschi et al., 2018).

During the pandemic, PTG has been documented in studies in several countries around the world including Spain (Vazquez et al., 2021), Israel (Hamam et al., 2021), Syria (El Khoury-Malhame et al., 2023), China (Zhen and Zhou, 2022), Japan (Okuyama et al., 2021), United States (Northfield and Johnston, 2022), and also countries in Latin America including Peru (Caycho-Rodríguez et al., 2023), and also in relation to post-traumatic stress disorder (PTSD) (Cénat et al., 2021; Chamaa et al., 2021: Ruiz-Frutos et al., 2021). Researchers explain that to experience post-traumatic growth, it is necessary to experience at least a moderate level of post-traumatic stress symptoms (Levine et al., 2008). This may explain why many studies have found significant associations between PTG and PTSD symptoms (see Liu et al., 2017 meta-analysis), including in COVID-19 studies (El Khoury-Malhame et al., 2023; Feingold et al., 2022; Jian et al., 2023).

Despite the association between PTS and PTG, these constructs are very distinct, and the paths explaining the development of each construct are quite different. For example, the development of PTS symptomatology and PTG will depend on aspects such as personality (e.g., neuroticism vs. agreeableness), coping strategies (e.g., avoidant coping vs. active coping), or social support (e.g., isolation vs. having contact with people and participating in social activities) among others (Boyraz et al., 2020; Henson et al., 2021; Kocjan et al., 2021; Shakespeare-Finch and Lurie-Beck, 2014; Taku and McLarnon, 2018; Taku et al., 2009). In consequence, when people can cope and adapt to distress (i.e., resilience), they may develop stress-related growth (Yıldırım and Arslan, 2021) including post-traumatic growth (Cárdenas et al., 2018). This was also the case during the COVID-19 pandemic, where positive associations between fear of COVID-19 and PTG were reported when mediated or moderated by coping and resilience (Fino et al., 2022a; Fino et al., 2022b; Yang et al., 2024; Yıldırım et al., 2022).

In psychology, psychological growth is a popular topic, especially when people can grow despite adversities. A psychological theory explaining psychological growth is self-determination theory (SDT; Ryan and Deci, 2017; Vansteenkiste and Ryan, 2013) which states that an individual’s psychological growth can be explained by the satisfaction of psychological needs (Deci and Ryan, 2000), which are defined as essential psychological nutrients for the adjustment, integrity, and wellbeing of individuals (Ryan, 1995; Ryan and Deci, 2017).

According to SDT, there are three basic psychological needs (autonomy, competence, and relatedness), which are innate, and each plays a fundamental role in optimal development so that none of these needs can be frustrated without experiencing several negative consequences, such as the risk of behavioral problems or psychopathology (Ryan and Deci, 2017; Vansteenkiste and Ryan, 2013).

The need for autonomy involves people’s efforts to be agents of their actions and decisions (volition), as well as to be able to determine their behavior (Ryan and Deci, 2017), and if this need is frustrated, the individual will feel controlled or forced to act in certain ways, against his or her will (Vermote et al., 2021). The need for relatedness involves the interest in relating to and caring (Ryan, 1995) where an individual needs to feel socially connected and to have a sense of belonging and that one is significant or important to others (Ryan and Deci, 2017; Šakan et al., 2020). When people experience relatedness, they show benevolence to others (2017n, 2017), but if this need is frustrated, rejection, loneliness, and disconnection from other people may be experienced (Vermote et al., 2021). Finally, the need for competence refers to feeling effective and skilled (Ryan and Deci, 2017). SDT posits that people need to feel efficacy in different important contexts of their lives (Ryan and Deci, 2017), and when this need is frustrated inefficacy and a decrease in personal confidence may be experienced (Spinelli et al., 2020).

SDT explains that individuals can grow even in dreadful circumstances such as the COVID-19 pandemic. During COVID-19, the three SDT needs were frustrated because (1) people experienced less autonomy, for example, by restricting the voluntariness of making people’s own decisions (e.g., restricting choices and prohibition leaving the house), by pressuring people, obliging them to do things (e.g., must wear masks all the time) (Vermote et al., 2021). (2) During the pandemic, physical contact with people was drastically reduced, physical contact with loved ones was reduced, family and group gatherings were prohibited, and in hospitals, people were not allowed to be with their loved ones (Lades et al., 2020). (3) Finally, the need for competence was also frustrated, many people were not able to pursue or fulfill specific goals, for example, many people had to perform many roles at the same time, and many doubted their abilities to balance and carry out different roles (such as parenting, working, and teaching), as well as the fact that various activities that offered the opportunity to perform well, and to develop new skills, were canceled, hindered the satisfaction of competence (Spinelli et al., 2020).

However, for many people, need satisfaction was possible, and a source of resilience, given that helped people reduce concerns in the face of adverse events and helped people to deal better with concerns serving as a coping mechanism (Waterschoot et al., 2023). For example, (1) many people found more time for developing new interests and hobbies (Güzel et al., 2020), (2) found a way to connect with other people using alternative ways (e.g., virtually), or began to participate more in collective activities that generate a sense of mutual support and belonging to a group (Lades et al., 2020), and (3) the pandemic also offered opportunities to acquire new skills and knowledge, as well as the opportunity to improve other skills that previously there was not as much time to practice (Güzel et al., 2020).

So, in times of uncertainty and insecurity, such as this pandemic, promoting the satisfaction of basic psychological needs may be essential to maintaining good mental health and wellbeing (Vermote et al., 2021; Waterschoot et al., 2023). With these in mind, it is possible to see how SDT’s need satisfaction will relate to PTG since its pillars are consistent with SDT’s basic needs (Tedeschi and Calhoun, 1996, 2004; Ryan and Deci, 2000; Ryan and Deci, 2017). For instance, PTG relating to others (greater sense of closeness with others) is consistent with SDT relatedness (meaningful interpersonal relations). PTG’s appreciation of life and spiritual change include changing priorities regarding what is important in life and having a better understanding of spiritual matters (respectively), which are both consistent with SDT’s need for autonomy which involves doing things that feel important to you, that are consistent with personal values (spiritual ones); and PTG’s personal strength (e.g., developing a greater feeling of self-reliance) and new possibilities (developing new interests, doing better things with one’s life) can relate to SDT’s need for competence, which involves doing things that you feel you are good at.

Several studies investigated whether basic psychological needs would play a role in wellbeing during the pandemic. For example, a study in the U.S. found that mental wellbeing during a pandemic was positively related to SDT’s basic psychological need (BPN) satisfaction, especially the need for relatedness, given the possibility to work having contact with people virtually or by telephone (Cantarero et al., 2021). In another study conducted in Serbia, with a sample of approximately 1,000 adult participants found that to be fully functional and promote wellbeing during the pandemic, all three basic psychological needs needed to be satisfied (Šakan et al., 2020). In addition, in one of the most comprehensive and extensive studies using SDT’s motivational framework, the Motivation Barometer (Vansteenkiste et al., 2024) evaluated the role of SDT’s need satisfaction and frustration in relation to COVID-19-related phenomena (e.g., vaccination, restrictions, and quality of life) and reported that psychological need fulfillment was a source of resilience against ill-being (Vermote et al., 2021; Waterschoot et al., 2023).

Despite the research involving SDT needs and COVID-19, there is little research on the relationship between BPNs and post-traumatic growth in the context of COVID-19. So, this research aimed to study the relationship between fear of COVID-19, basic psychological needs (satisfaction and frustration), post-traumatic growth, and post-traumatic symptomatology in Peruvian adults. Considering the theoretical background of this study, it is possible to expect that fear of COVID-19 will be positively associated with PTG, and this association will be mediated by the satisfaction of BPNs. In addition, it is possible to expect that fear of COVID-19 will have a positive association with PTS symptomatology, and this relationship will be mediated by the frustration of BPNs. In addition, because it is the first time these constructs have been studied in Peru regarding COVID-19, we will also explore the differences between sexes (men and women).

## Methods

### Participants

The study sample consisted of 361 Peruvian adults (70.6% women, *N* = 253; 29.2% men, *N* = 108). The mean age of the participants was 35.04 years (*SD* = 16.55), ranging from 18 to 84 years old. In addition, 299 (82.8%) participants said to have had physical contact with people outside the home, and 345 (95.6%) said to have had virtual contact with people outside their home. In addition, 107 (29.6%) reported not doing any exercise, 84 (23.3%) reported exercising 1 or 2 times a week, 84 (23.3%) reported exercising 3 times a week, and 86 (23.8%) reported exercising four more times a week. A big majority (*n* = 357, 98.9%) reported having or knowing people who has or had COVID-19. This study was performed in the first year of the pandemic during lockdown.

### Materials

*Sociodemographic information*. A data sheet was created asking information about age, sex (0 = Male, 1 = Female), country of birth, having contact with other people outside the home either virtually and/or physically (0 = not having, 1 = having), frequency of exercising (0 = not exercising, 1 = 1 or 2 times a week, 2 = 3 times a week, 3 = 4 times a week or more), and whether they, or people they know, have or had COVID-19 (0 = not having, 1 = having).

*Fear of COVID-19 Scale (FCV-19S)* (Ahorsu et al., 2020): This seven-item scale measures the severity of people’s fear of COVID-19 from 1 (strongly disagree) to 5 (strongly agree), so higher scores show higher fear of COVID-19. This scale has presented good psychometric properties in the original study (Ahorsu et al., 2020) and in Peru (Hernández et al., 2021; Huarcaya-Victoria et al., 2020). Example items are “I am most afraid of coronavirus-19” or “It makes me uncomfortable to think about coronavirus-19.” In the present research, factor analysis showed a single factor and good internal consistency (Cronbach’s alpha = 0.85).

*Impact event scale-revised (IES-R)* (Weiss and Marmar, 1997): This scale is intended to measure the subjective PTS caused by a specific traumatic event (in the last week) from 0 (“no or never”) to 4 (“yes, extremely”), so higher scores show higher PTS. This 22-item scale measures three groups of symptoms consistent with DSM-IV: intrusion, avoidance, and hyperarousal, that has been put together in COVID-19 research in a composite severity score (Terzioğlu and Büber, 2021; Andhavarapu et al., 2022). The scale showed good psychometric properties in Peru (Gargurevich et al., 2009). In the present research, items were adapted to measure COVID-19 (“I have tried not to talk about COVID-19”; “I have thought about COVID-19 without intending to”). In the present study, the scale showed a single factor with good internal consistency (Cronbach’s alpha = 0.93).

*Basic psychological need satisfaction and frustration scale (BPNSFS)* (Chen et al., 2015): This scale of 24 items aims to measure satisfaction and frustration of the three basic psychological needs of autonomy, relatedness, and competence using a 7-option Likert-type scale (from “completely false” to “completely true”), so higher scores show higher levels of need satisfaction and higher levels of need frustration. The Spanish version of the scale showed good psychometric properties in Peru (Chen et al., 2015). This scale can assess each need independently but also jointly in two factors (need satisfaction and frustration) as showed by bifactor studies studying the scale (Tóth-Király et al., 2018; Garn et al., 2019). Some examples of items are “I feel that I have been doing what really interests me” (autonomy satisfaction) or “I have serious doubts about whether I can do things well” (competence frustration). In the present study, factor analysis showed two factors: need satisfaction and need frustration [*x*^2^(251) = 403.39 (*p* < 0.001), RMSEA = 0.041 (90% CI: 0.034–0.048), SRMR = 0.073, CFI = 0.99] with factor loadings ranging from 0.76 to 0.43 and 0.72 to 0.56 for factors one (need satisfactions) and two (need frustration), respectively. In addition, factors one and two obtained good internal consistency (Cronbach’s alpha of 0.89 and 0.90, respectively).

*Post-traumatic growth inventory (PTGI)* (Tedeschi and Calhoun, 1996): This 21-item scale aims to measure post-traumatic growth in people after a traumatic event through five subscales (interpersonal relationships, appreciation for life, new possibilities, personal strength, and spirituality) from 0 (did not experience this change) to 5 (I experienced this change to a great degree), so higher scores show higher PTG. The scale had good psychometric properties in the original study (Tedeschi and Calhoun, 1996) and in Peru (Gargurevich, 2016). For this research, the scale’s instructions were adapted for COVID-19 as the traumatic event item examples: “I changed my priorities about what is important in life” or “I have developed new interests.” These scales can be collapsed together into a PTG global score (Gori et al., 2021; Ulset and von Soest, 2022). In the present study, the scale showed a single factor with good internal consistency (Cronbach’s alpha = 0.95).

### Procedure

All questionnaires were applied online via Google Forms. Participants were summed via social media (e.g., Facebook and Instagram). Before answering the questionnaires, all participants completed an informed consent making clear issues of anonymity, voluntary participation (no compensation) and the option to withdraw at any time without this being detrimental in any way. Several ethical criteria were considered, such as the criterion of rigorousness as transparency, for which it was explained in the informed consent in detail about the questionnaire and research as well as credibility, for which a written return of the results was stated for those who want it.

### Data analysis

Psychometric analyses as well as descriptive and correlational analyses were performed using the Statistical Package for Social Sciences (SPSS v.28; IBM Corp, 2021). Evidence of factorial validity and reliability (Cronbach’s alpha) was first performed. Next, skewness and kurtosis were performed to explore the normal distribution of the data. Then, descriptive and correlation analyses were performed between the studied variables.

Finally, path analysis was carried out to explore the hypothesized relations between the study variables using the Linear Structural Relations (LISREL) program version 8.5 (Jöreskog and Sörbom, 1996). To evaluate the fit of the model, the chi-square, the comparative fit index (CFI), the root mean square error of approximation (RMSEA), and the standardized root mean square residual (SRMR) were used. To evaluate the suitability of the model, the combination of various fit indices was used. In this way, indices such as chi-square (*X*^2^), root mean square of approximation (RMSEA), standardized root mean square residual (SRMR), and comparative fit index (CFI) were used. Thus, it is expected that, after calculating the various models, the most appropriate one will have the lowest possible chi-square, values close to 0.06 in the RMSEA and 0.08 in the SRMR, as well as scores ≥0.95 in the CFI (Hu and Bentler, 1999). Also in case of competing models, the Akaike information criterion (AIC) was used given that the lower AIC shows the more parsimonious model (Chakrabarti and Ghosh, 2011).

## Results

In Table 1, means, standard deviations, and correlations between the studied variables can be seen. Asymmetry and kurtosis were calculated, and values were lower than |2| meaning that the distribution of the variables resembles a normal one (George and Mallery, 2010).

**Table 1:** Means (M), standard deviations (SD), and correlations between studied variables.

Measures	*M*	*SD*	Age	Sex	Physical contact	Exercise	1	2	3	4
1. Fear of COVID-19	2.30	0.84	−0.01	0.23**	−0.12*	−0.01	一			
2. Need satisfaction	4.23	0.60	0.23**	0.05	0.11	0.23**	−0.16**	一		
3. Need frustration	2.17	0.79	−0.30**	−0.00	0.04	−0.16**	0.35***	−0.67***	一	
4. PTS symptoms	1.38	0.81	−0.19**	0.18**	−0.05	−0.02	0.67***	−0.27***	0.51***	一
5. Post-traumatic growth	3.28	1.09	0.04	0.14**	0.06	0.07	0.22***	0.30***	−0.04	0.26***

*p < 0.05, **p < 0.01, and ***p < 0.001; PTS, post-traumatic stress symptom.

### Descriptive analyses

From the demographic variables, age, sex, having psychical contact, and exercising correlated significantly with a psychological variable. Age correlated positively with need satisfaction and negatively with need frustration and with PTS symptoms. Sex had a point biserial positive correlation with fear of COVID-19, PTS symptoms, and PTG, showing that men (*M_men_* = 2.01, *SD* = 0.74) reported less fear of COVID-19 than women (*M_women_* = 2.42, *SD* = 0.85), reported (*M_men_* = 1.15, *SD* = 0.72) less PTS symptoms than women (*M_women_* = 1.47, *SD* = 0.83), and reported less PTG (*M_men_* = 3.04, *DE* = 1.14) than women (*M_women_* = 3.38, *DE* = 1.05). In addition, having physical contact with someone outside the home had a significant point biserial correlation with fear of COVID-19, so those not having physical contact with someone outside the home reported higher values of fear of COVID-19 (*M_not contact_* = 2.52, *SD* = 0.98) than the ones having contact outside their homes (*M_contact_* = 2.25, *SD* = 0.80). In addition, exercising correlated positively with need satisfaction and negatively with need frustration.

### Correlation analyses

Regarding the psychological variables, fear of COVID-19 correlated negatively with need satisfaction and positively with need frustration, PTS symptoms, and PTG (although in lower intensity than the correlation with PTS symptoms). Need satisfaction correlated positively with PTG and negatively with need frustration and PTS symptoms, and need frustration correlated positively with PTS symptoms and had no significant correlation with PTG. Finally, PTS symptoms correlated positively with PTG (see Table 1).

Before calculating the path analysis models, a partial correlation was performed between fear of COVID-19, PTG, and PTS to observe the real association between these variables. The results showed that when correlating fear of COVID-19 and PTG, controlling for PTS symptoms, the correlation was not significant (*r* = 0.07, *p* = 0.19), and when correlating fear of COVID-19 with PTS symptoms controlling for PTG, the correlation was still significant (*r* = 0.65, *p* < 0.001) as in the zero-order correlation. This is particularly important for building the path models because the estimation of the paths is based on the significant correlations among the studied variables, hoping not to have spurious correlations like it seem to be happening between fear of COVID-19 and PTG in the zero-ordered correlation.

### Path analysis

To analyze the relationship between the variables proposed by this research, a path analysis was carried out. All analyses were performed using maximum likelihood given that variables were normally distributed. Despite the hypothesized full mediation model (analyzing the paths from fear of COVID-19 to post-traumatic growth or post-traumatic symptomatology through the satisfaction or frustration of BPNs), the path analyses allowed us to analyze also the direct effects and the cross-path effects between the variables. So, to check the relationships between the variables, three models were carried out. Associations of variables within the models were modeled after the significant correlations obtained between the variables. Models were controlled for sex, age, having physical contact with someone outside the home, and the frequency of exercising, and in all models, the correlations between need satisfaction and frustration and between PTG and PTS symptoms were calculated.

In the first model (Model 1), a total mediation model was carried out in which the bright and dark sides could be clearly represented. Thus, in this model, need satisfaction mediated the relationship between fear of COVID-19 and post-traumatic growth, while need frustration mediated the relationship between fear of COVID-19 and post-traumatic symptomatology. This model did not achieve good fit indices: *x*^2^(15) = 159.20 (*p* < 0.001), RMSEA = 0.16 (90% CI: 0.14–0.19), SRMR = 0.087, CFI = 0.78, and AIC = 219.20. So, a second model was calculated (Model 2).

In Model 2, in addition to including the effects of Model 1, the direct effect of fear of COVID-19 to PTS symptomatology was added (the path from fear of COVID-19 to PTG was not included given the lack of correlation between these variables after the partial correlation). This model obtained acceptable to good fit indices: x ^2^(14) = 51.67 (*p* < 0.001), RMSEA = 0.083 (90% CI: 0.062–0.11), SRMR = 0.059, CFI = 0.95; and AIC = 113.67. Finally, in Model 3, the effects of Model 2 were considered, and the direct crossed effect from need satisfaction to post-traumatic symptomatology was added (the effect from need frustration to post-traumatic growth was not added due to the lack of correlation between these variables). This model also obtained good fit indices, but the RMSEA was not acceptable [ x ^2^ (13) = 51.16 (*p* < 0.001), RMSEA = 0.091 (90% CI: 0.065–0.12), SRMR = 0.059, CFI = 0.95, and AIC = 115.16]. So, of the three models, Model 2 was the best fitting model since it obtained fit indices between good and acceptable in all cases (also, the AIC value was lower in Model 2).

**Figure 1:**
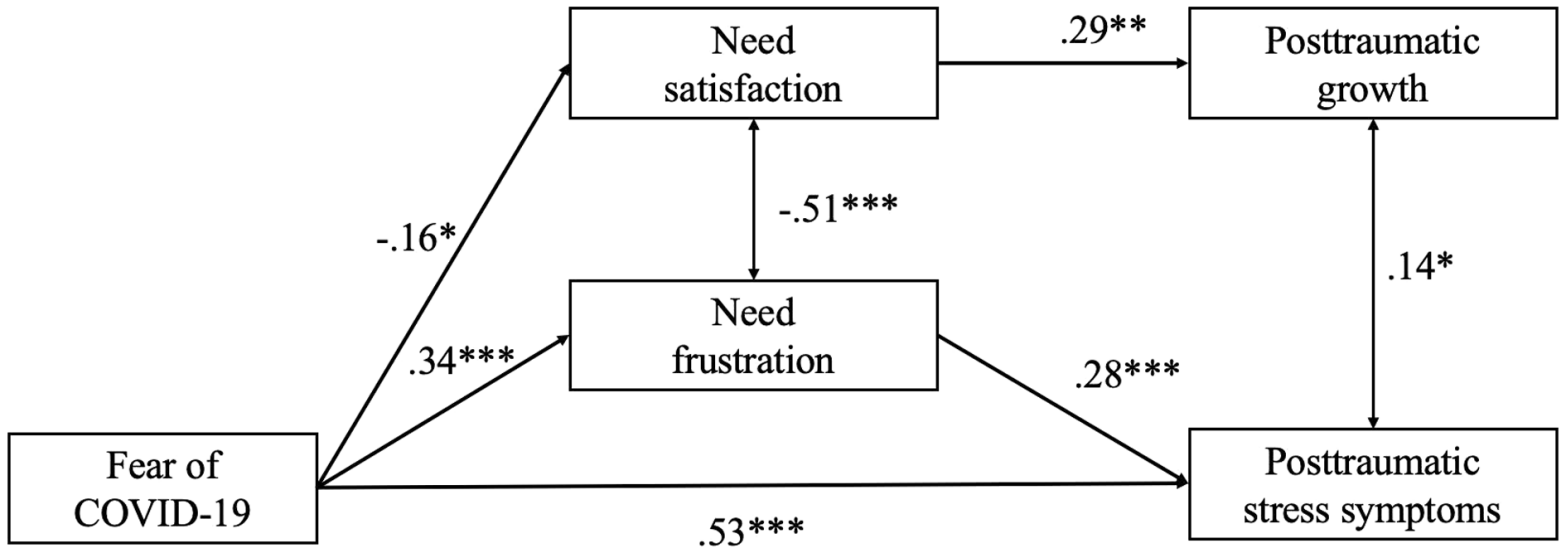
Model 2 path analysis. *p < 0.05, **p < 0.01, ***p < 0.001.

Paths from Model 2 can be seen in Figure 1. In the figure, it is possible to see that fear of COVID-19 negatively predicted PTG through the mediation effect of need satisfaction and positively predicted PTS symptoms through the mediation effect of need frustration. In addition, there is a positive direct effect from fear of COVID-19 to PTS symptoms. It can be seen in the figure that the total effect of fear of COVID-19 on PTG is much lower (−0.05, *R^2^* = 0.11) than the effects on PTS (0.63; *R^2^* = 0.53%).

## Discussion

The COVID-19 pandemic changed people’s life in multiple ways. Suddenly, people needed to change their daily routine (e.g., go shopping for basic goods, work online from home, and washing hands several times a day), and people were not allowed to socialize (e.g., meetings with friends and family were prohibited). At the beginning of the pandemic, the unknowns about the illness were many, and many people were hospitalized and died. Given the many deaths in Peru, fear of dying by the virus created great distress among Peruvians (Ruiz-Frutos et al., 2021), so fear of COVID-19 was a logical response (Luo et al., 2021).

People need to adapt to the challenges posed by the pandemic, and it is important to understand the mechanism that may lead individuals to experience distress or to be resilient and experience psychological growth. This is why the present research studied the relationship between fear of COVID-19, basic psychological needs, post-traumatic growth, and post-traumatic symptomatology in Peruvian adults. Need satisfaction has been stated as an important aspect conducing to wellbeing while need frustration to ill-being (Ryan and Deci, 2017). This was found during the COVID-19 pandemic as BPN’s satisfaction and frustration mediated associations between COVID-19 insecurity and wellbeing and ill-being, respectively (Vermote et al., 2021).

Given the theoretical information, it was hypothesized that BPN satisfaction would mediate the positive relationship between fear of COVID-19 and PTG, and BPN frustration would mediate the positive relationship between fear of COVID-19 and PTS symptomatology. This hypothesized model was studied on a sample of 361 Peruvian adults in the first year of the COVID-19 pandemic (during lockdown), and the results confirmed our hypothesis partially. That is both BPN satisfaction did mediate the association between fear of COVID-19 and PTG, but this association was negative (and not positive as hypothesized). BPN frustration significantly mediated the positive association between fear of COVID-19 and PTS symptoms, and a significant main effect from fear of COVID-19 to PTS symptoms was found.

It is interesting to highlight that in the present study, fear of COVID-19 positively correlated with PTG and PTS symptoms, and there was also a positive correlation between PTG and PTS. This might be happening because both originated after the same event; however, they are different psychological phenomena. Correlations between fear of COVID-19 and PTG have been shown in previous research (Fino et al., 2022a; Fino et al., 2022b; Yang et al., 2024) as well as with PTS (Şimşir et al., 2022); however, in the present results, after controlling for the shared variance with PTS, the correlation between fear of COVID-19 and PTG disappeared.

The results also showed sex differences on fear of COVID-19, PTS, and PTG. In the present research, women reported experiencing more fear of COVID-19 (Andrade et al., 2022; Broche-Pérez et al., 2022a, 2022b), more PTS (Liu et al., 2020; Wang et al., 2020), and more PTG (Vishnevsky et al., 2010; Cohen-Louck, 2022) than men. It seems that despite women experiencing more PTS symptoms, women in Peru were more likely to follow government public health recommendations than men (Carreras et al., 2022), engaging in more self-reflection (Prieto-Ursúa and Jódar, 2020), a key aspect of PTG, and were able to find positive meaning in home and family-related activities during lockdown (Cohen-Louck, 2022).

The present results show that a stressful phenomenon, such as fear of COVID-19, can generate different psychological responses in people (Harper et al., 2021) and in combination with other factors, a positive or negative response may occur (Płomecka et al., 2020). So, in the present study, BPNs have an important role as mediators, since depending on whether these needs are satisfied or frustrated, the psychological result is different. These results support theoretical and empirical evidence that suggests that BPN satisfaction in times of COVID-19 is essential to maintaining good mental health and wellbeing (Vermote et al., 2021; Waterschoot et al., 2023), but it is important to note that despite the significant association between fear of COVID-19 and PTG, the effect through need satisfaction was lower in comparison with the effects when need frustration was the mediator, demonstrating that need frustration experiences played an important role in explaining ill-being in COVID-19 times. Interestingly, in addition to the effects through need frustration, there was a strong main effect on PTS symptoms (higher than the ones explained by the mediators). This is understandable because fear is an important aspect of PTS, as it appears as a persistent emotional state (American Psychiatric Association, 2013) involved in reexperiencing a traumatic event (Zoellner et al., 2020) and in avoidant behavior (López-Martínez et al., 2014; Sanchez-Gomez et al., 2021).

In addition, the present research collaborates with the literature looking into the importance of SDT’s basic psychological needs in non-WEIRD countries (like Peru), usually underrepresented in the international literature. Peru is considered highly collectivistic (Hofstede, 2001; Hofstede Center, 2024), giving great importance to social relations, so it is very interesting that need satisfaction (including autonomy and competence) can stand as a significant mediator in this context. So, the present results are consistent with SDT research claiming the universality of basic psychological needs (Ryan and Deci, 2000; Ryan and Deci, 2017).

### Limitations and implications

The present research results need to be considered at the light of some limitations. First, this is a cross-sectional study. Cross-sectional designs are very useful when explaining associations between variables especially when, for analytical purposes, these associations are placed as inputs and outputs guided by psychological theory, but it is important to remember that these associations are not causal inferences (Rindfleisch et al., 2008), and so it is important to be careful interpreting correlations results (Kesmodel, 2018). Further research may need to consider a longitudinal approach to study the joint development of psychological growth and distress, along with other intervening variables that have mediated-moderated COVID-19 growth and distress such as resilience or personal growth initiative (Green and Yıldırım, 2022), along with SDT’s psychological needs.

In addition, the generalizability of the results needs to be considered. Our sample is in no way a representative sample of Peruvians. Further studies may need to consider demographic aspects so as to have a representative sample. It is also important to mention that the present research focused on the experience of fear as a predictor of PTS, but many aspects related to COVID-19 (e.g., social restriction) were also associated with distress and not only fear (Guo et al., 2021; Matos et al., 2021; Rehman et al., 2023). Future research may consider assessing other COVID-19 stress-related phenomena (e.g., social isolation and using masks) and their impact on the life of Peruvians.

The present results may help in the development of SDT’s interventions in the PTS-PTG field. Successful SDT’s interventions have been developed in education and sports (Reeve et al., 2022; Raabe et al., 2019), and although more research is needed, the present results along with previous research (Vermote et al., 2021; Waterschoot et al., 2023) may give light into the role of need satisfaction as a resilience factor in stressful situation context (like in COVID-19).

## Conclusion

The results of the present research shed light on the association between fear of COVID-19, PTG, and PTS symptoms, considering the mediating role of SDT’s BPNs in Peru. The present results confirm the important role of BPN need satisfaction for wellbeing (PTG) and BPN frustration for ill-being (Ryan and Deci, 2017). In this way, the present results can be used as a steppingstone for studies analyzing distressing or trauma-related variables such as PTS and PTG considering using SDT’s BPN satisfaction and frustration as mediators.

## Data Availability

The raw data supporting the conclusions of this article will be made available by the authors, without undue reservation.
